# Supplementary material for: Phenolic Compounds in Berries of Winter-Resistant Actinidia arguta Miq. and Actinidia kolomikta Maxim.: Evidence of Antioxidative Activity
Source: Antioxidants (Basel). 2024 Mar 19;13(3):372. doi: 10.3390/antiox13030372 (PMC10968433; doi:10.3390/antiox13030372)
Supplement: Supplementary file 1 [file antioxidants-13-00372-s001.zip › antioxidants-2862518-supplementary.pdf]

Table S1. HPLC-MS/MS retention time and calibration data for phenolics.

Compound	Figure	Retention time, min (standard)	Retention time, min (sample)	Calibration curve	Coefficient of determination (r^2)
Neochlorogenic acid	S1	2.68	2.74	132.157x-36.1651	0.996665
Kaempherol-3-*O*-rutinoside (Nictoflorin)	S1	5.67	5.72	104.758x-25.1861	0.999235
Quercetin 3-*O*-glucoside (Isoquercitrin)	S1	5.49	5.53	371.928x+61.7969	0.991011
Luteolin-4-*O*-glucoside (Juncein)	S1	5.89	5.92	701.516x+148.51	0.986978
Luteolin-7-rutinoside (Scolymoside)	S1	5.55	5.59	362.482x-38.6976	0.998807
Procyanidin B1	S1	3.55	3.59	24.889x-10.2825	0.987535
Procyanidin C1	S1	4.6	4.65	31.3648x-7.97714	0.999019
(+)-Catechin	S2	3.81	3.85	7.65941x-1.7829	0.985683
Chlorogenic acid	S2	3.91	3.94	123.701x-15.508	0.992902
Phloridzin	S2	6.07	6.12	1024.59x+15.3622	0.998189
Quercetin	S2	7.05	7.09	408.567x-184.72	0.996974
Isorhamnetin-3-*O*-rutinoside (Narcissin)	S2	5.75	5.78	513.81x-36.5168	0.999203
Ferulic acid	S2	5.45	5.49	135.218x-13.05	0.994858
Procyanidin B2	S2	4.17	4.2	83.8582x-12.343	0.996484
(−)-Epicatechin	S3	4.37	4.44	8.52997x-2.9562	0.996997
Caffeic acid	S3	4.17	4.23	36.2607x+1.12917	0.990083
Kaempherol-3-*O*-glucoside (Astragalin)	S3	5.87	5.92	418.643x+63.4945	0.998666
Quercetin-3-*O*-rutinoside (Rutin)	S3	5.31	5.35	209.907x-11.322	0.998333
Quercetin-3-*O*-galactoside (Hyperoside)	S3	5.44	5.48	366.477x-121.168	0.99818

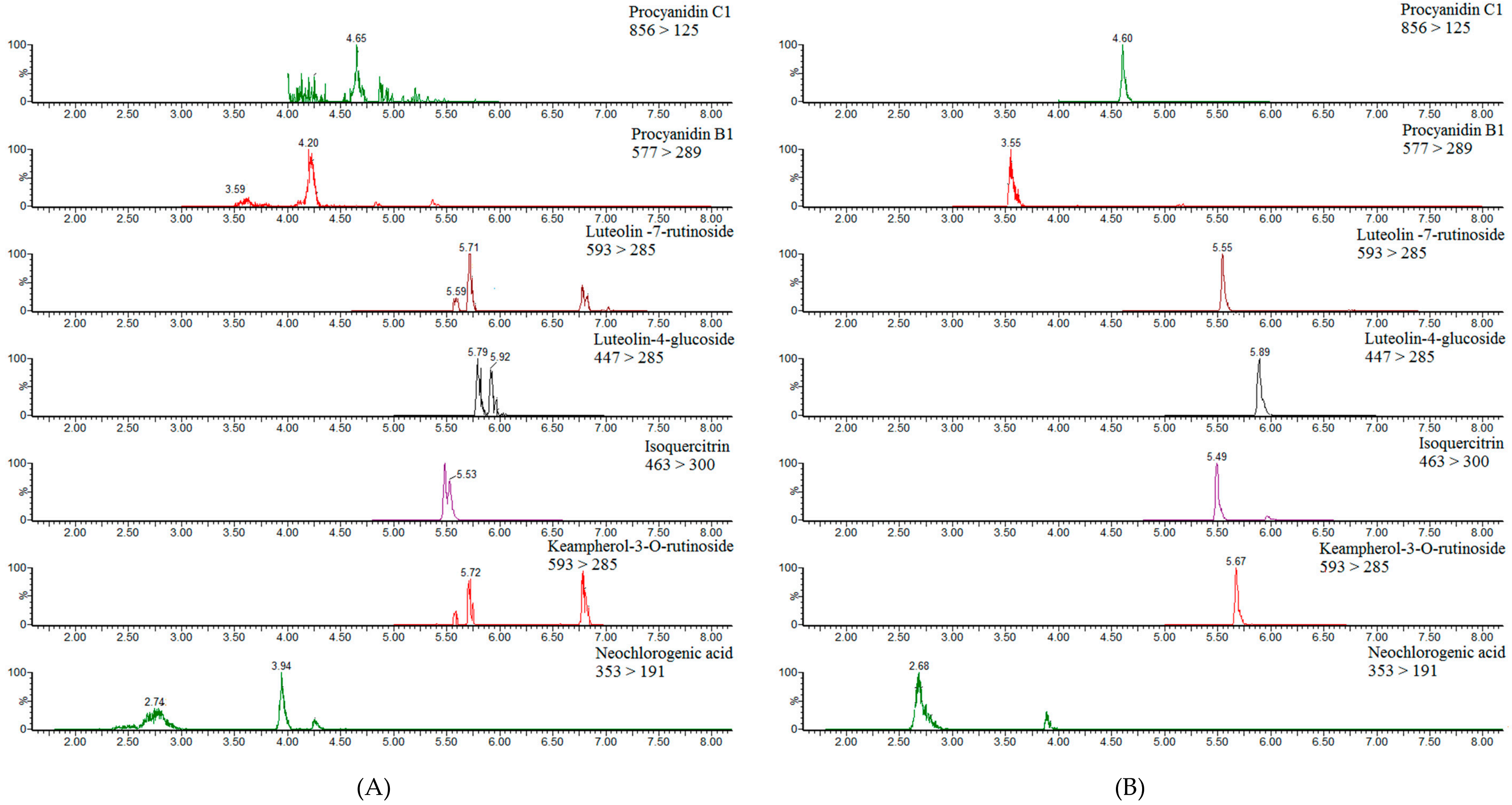

Figure S1. MS/MS chromatograms of *A. kolomikta* (A) and phenolic compounds standard mixture (B): procyanidin C1, procyanidin B1, luteolin-7-rutinoside, luteolin-4-*O*-glucoside, isoquercitrin, kaempferol-3-*O*-rutinoside, and neochlorogenic acid.

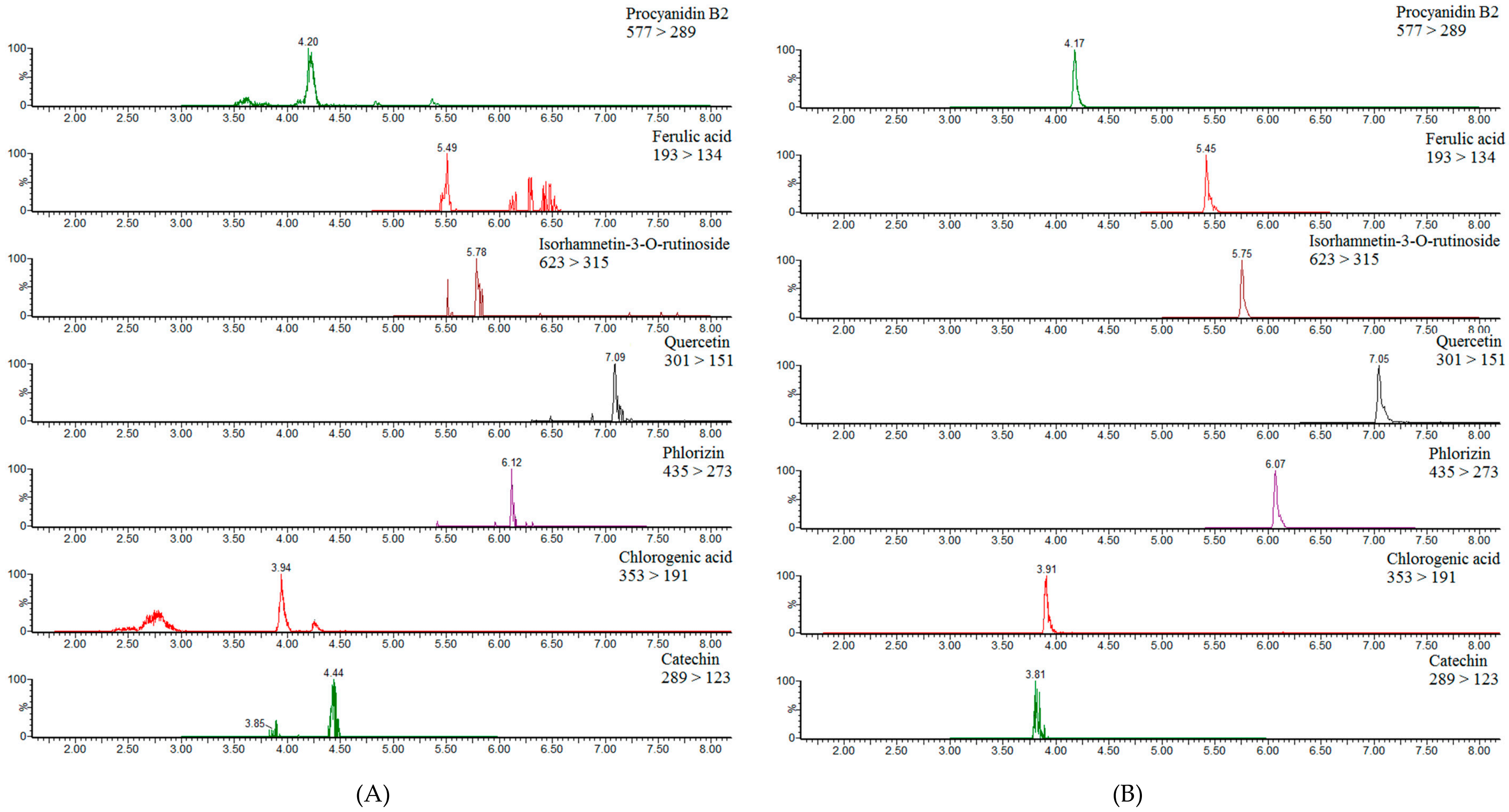

Figure S2. MS/MS chromatograms of *A. kolomikta* (A) and phenolic compounds standard mixture (B): procyanidin B1, ferulic acid, isorhamnetin-3-*O*-rutinoside, quercetin, phlorizin, chlorogenic acid, and catechin.

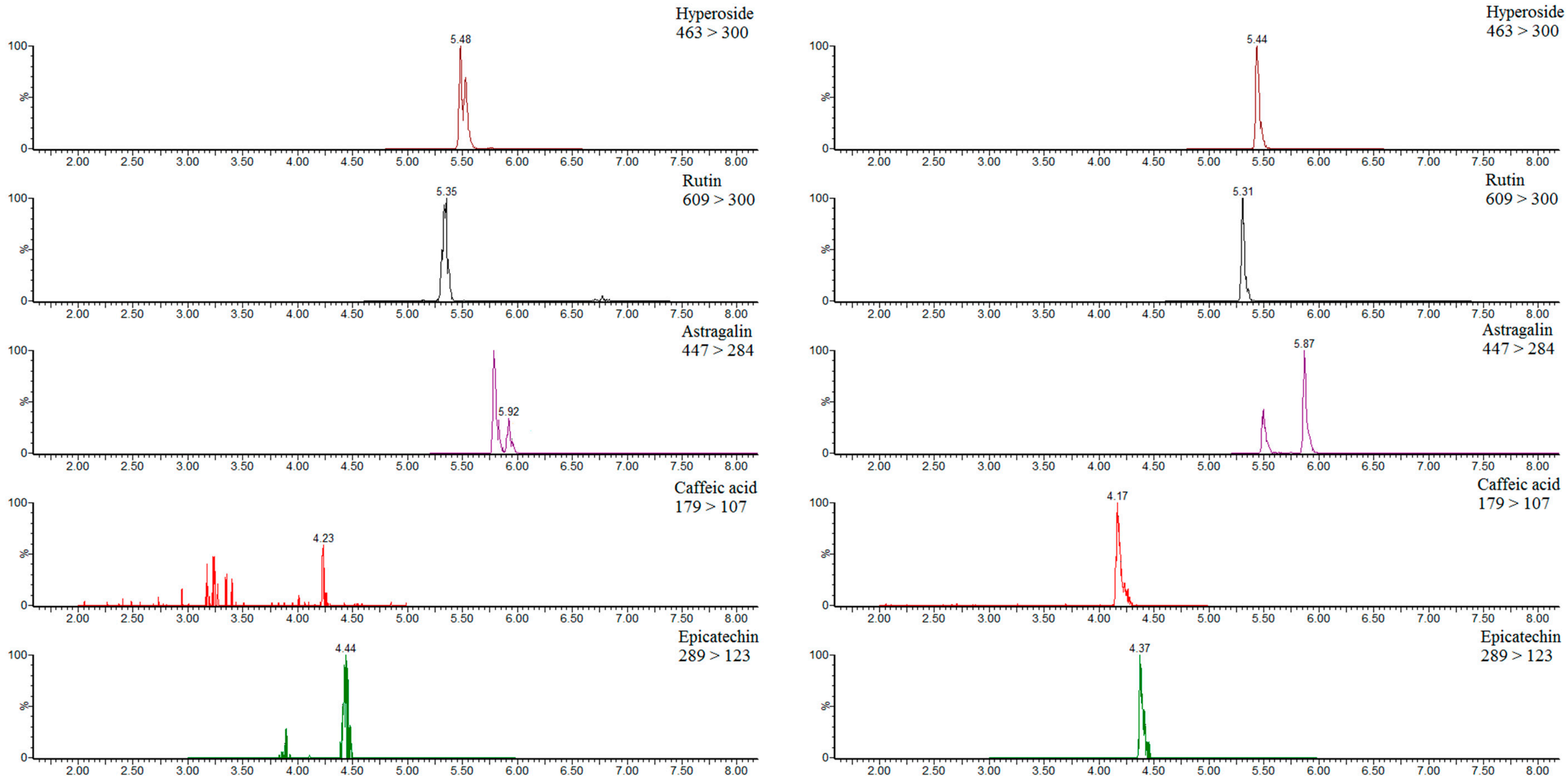

(A) (B)

Figure S3. MS/MS chromatograms of *A. kolomikta* (A) and phenolic compounds standard mixture (B): hyperoside, rutin, kaempferol-3-*O*-glucoside, caffeic acid, and epicatechin.
